# The Impact of Avian Haemosporidian Infection on Feather Quality and Feather Growth Rate of Migratory Passerines

**DOI:** 10.3390/ani14121772

**Published:** 2024-06-12

**Authors:** Carlos Mora-Rubio, Luz Garcia-Longoria, Martina Ferraguti, Sergio Magallanes, João T. Cruz, Florentino de Lope, Alfonso Marzal

**Affiliations:** 1 Department of Anatomy, Cellular Biology and Zoology, University of Extremadura, 06006 Badajoz, Spain; luzlongoria@unex.es (L.G.-L.); fdelope@unex.es (F.d.L.); amarzal@unex.es (A.M.); 2 Department of Conservation Biology and Global Change, Doñana Biological Station (EBD), Consejo Superior de Investigaciones Científicas (CSIC), 41092 Sevilla, Spain; mferraguti@ebd.csic.es (M.F.); sergio.magallanes@ebd.csic.es (S.M.); 3 Centre for Biomedical Research in Epidemiology and Public Health Network (CIBERESP), Madrid, Spain; 4 Centre for Interdisciplinary Research in Animal Health (CIISA), Faculty of Veterinary Medicine (FMV-ULisboa), University of Lisbon, 1300-477 Lisbon, Portugal; joaot99fmv@gmail.com; 5 Associate Laboratory for Animal and Veterinary Sciences (AL4AnimalS), 1300-477 Lisbon, Portugal; 6 Wildlife Research Group, San Martin National University, 22021 Tarapoto, Peru

**Keywords:** feather growth rate, feather quality, *Haemoproteus*, haemosporidian parasites, *Leucocytozoon*, moult, *Plasmodium*, uropygial gland

## Abstract

**Simple Summary:**

Feathers are essential for a bird’s flight, insulation, communication, and camouflage. They degrade over time, so birds must moult regularly. This study examined how avian haemosporidian infection and the size of the uropygial gland affect feather quality and growth rate in two migratory bird species in southwestern Spain—the house martin (*Delichon urbicum*) and the sand martin (*Riparia riparia*). We found that house martins had the highest haemosporidian infection rates, likely due to their large colony size. Infection only decreased feather quality in house martins and did not affect the feather growth rate in any of the two hirundinids. Additionally, feather growth rate was positively linked to feather quality, but only in house martins. Finally, we found no connection between the uropygial gland size and feather quality or feather growth rate. These results show, for the first time, that avian haemosporidian parasites can negatively impact the feather quality of migratory birds, thus potentially affecting their flight and survival. Further research is needed to fully understand these relationships.

**Abstract:**

Bird feathers have several functions, including flight, insulation, communication, and camouflage. Since feathers degrade over time, birds need to moult regularly to maintain these functions. However, environmental factors like food scarcity, stress, and parasite infections can affect feather quality and moult speed. This study examined the impact of avian haemosporidian infection and uropygial gland volume, as well as feather quality and feather growth rate in two migratory hirundine species captured in southwestern Spain—the house martin (*Delichon urbicum*) and sand martin (*Riparia riparia*). Our findings showed that the prevalence of infection varied among species, with house martins having the highest rates, possibly due to their larger colony size. Moreover, haemosporidian infection had a different impact on each species; infected house martins exhibited lower feather quality than healthy individuals, although this outcome was not observed in sand martins. Furthermore, no effect of infection on feather growth rate was observed in both hirundinids. Additionally, feather growth rate only correlated positively with feather quality in house martins. Finally, no link was observed between uropygial gland volume and feather quality or feather growth rate in any of the species in this study. These findings highlight the effect of haemosporidian infections on the plumage of migratory birds, marking, for the first time, how avian haemosporidian infection is shown to adversely impact feather quality. Even so, further research is needed to explore these relationships more deeply.

## 1. Introduction

Feathers are essential for birds, greatly assisting them in flight by covering the wings with aerodynamically efficient structures and forming an aerofoil-shaped body [1]. The flight-oriented feathers of the wings are called remiges, which are divided into primaries, secondaries, and tertiaries; those that cover the gaps at the base of remiges are termed coverts or tectrices. Also, tail feathers are typically flight-oriented as well and are called rectrices [1]. Besides locomotion, feathers also perform other critical functions in birds (see review in [2]). For example, the colour and shape of plumage largely determine the appearance of birds, which is intimately linked to camouflage and to both intra- and interspecific communication [3]. In addition, modifications on the structure of feathers aid in buoyancy and waterproofing, hence allowing many groups of birds to inhabit aquatic and marine environments [4,5]. Moreover, some specialized feathers in facial discs can help some Strigiformes to amplify and localize sounds in total darkness, improving their hunting capabilities [6].

However, feathers are lost or continuously damaged due to mechanical abrasion, alterations of the physical structure of keratin during sunlight exposure, ectoparasites consuming feather material, or keratin-degrading bacteria [7,8,9]. With the aim of keeping the plumage in optimal condition and maintaining its functionality, worn feathers need to be periodically replaced, a process termed moulting. Because new feathers are fully developed and functional long after old feathers are shed, birds unavoidably experience a temporary reduction in plumage function during the moulting period [1], which may impair their survival due to increase predation vulnerability [10], reduction in aerobic scope [11], and/or a decrease in thermoregulatory capabilities [7]. Hence, the fast replacement of feathers would minimize the time of reduced plumage functionality. As moult duration is linked to the feather growth rate [12], selection might therefore favour birds with increased feather growth rate. This could be especially relevant in bird species showing time constraints in their annual cycle, such as long-distance migrants, which may accelerate the moult by increasing the growth rate of individual feathers [1].

Feather quality is also critical for bird fitness, due to its close relationship with feather abrasion resistance [13], which is crucial for intensive locomotor activities such as long-distance migration. In this sense, it has been proposed that long-distance migrants probably suffer more feather wear, particularly due to sunlight [1]. It has been demonstrated that feathers of suboptimal quality often led to reduced efficiency in flight, foraging, and mating [14,15,16,17]. Moreover, the quality of feathers, including their strength, flexibility, and resistance to wear, is pivotal for the survival and reproductive success of birds [18]. Furthermore, poor-quality feathers may show a weakened feather structure that can lead to feather breakage [19], which may impair efficient escape from predators [20]. Also, [21] revealed that the flight performance of the trans-Saharan migrant barn swallows (*Hirundo rustica*) is negatively associated with the number of holes in the wings’ flight feathers, which is one of the main indicators of the functional quality of these feathers [22].

Bird malaria and related haemosporidian parasites (genera *Plasmodium*, *Haemoproteus,* and *Leucocytozoon*) are a diverse group of protozoans with widespread global distribution that infect bird species from a wide range of taxonomic orders [23,24]. Their life cycles are obligately heteroxenous, comprising sexual reproduction stages within the dipteran vector, whereas asexual reproduction takes place on a vertebrate host [23,25]. Because of their diversity, abundance, and wide geographical distribution, empirical and experimental studies on avian malaria and related haemosporidians nowadays provide a distinctive animal model for comprehending the ecology and evolution of vector-borne diseases [26]. An infection with haemosporidian parasites provokes detrimental effects on several traits of their avian hosts, resulting in reduced survival [27,28,29,30], impaired body condition [31,32], and decreased reproductive success [33,34], among others. However, the negative impact of avian haemosporidians on some other host traits has not yet been addressed. For example, although some studies have analysed whether parasites may provoke adverse effects on bird plumage [35,36,37], the number of studies exploring the effects of haemosporidian infection on the feather growth rate and feather quality of birds is still limited to a reduced number or species, and the results remain inconclusive [38,39,40,41]. For instance, Fithian [38] explored the relationship between haemosporidian infection and feather reflectance in adult prothonotary warblers (*Protonotaria citrea*). While no significant correlation was found between infection status and visible light reflectance or hue, haemosporidian-infected birds tended to exhibit lower levels of UV reflectance. Also, Marzal et al. [39] revealed a detrimental association between avian haemosporidian infection and the inferred growth rate of the tail feathers of house martins (*Delichon urbicum*), showing a lower feather growth rate in haemosporidian-infected birds. Moreover, Marzal et al. [40] also found that house martins harbouring co-infections with two haemosporidian lineages exhibited the lowest inferred growth rate in their tail feathers when compared to uninfected and single-infected individuals, although no effect of haemosporidian co-infection was observed on the feather quality of birds. Additionally, they found a negative correlation between feather quality and feather growth rate, suggesting a trade-off between both traits. Furthermore, Coon et al. [41] inoculated *Plasmodium* in house sparrows (*Passer domesticus*), experimentally demonstrating that haemosporidian infection reduced the feather growth rate.

The uropygial gland (also named *preen gland*) is a holocrine gland located in the integument above the posterior free caudal vertebrae of most bird species [42]. Uropygial gland secretion is mainly composed of a wide variety of substances, such as waxes, alcohols, terpenes, and fatty acids [43]. Among other functionalities, it has been proposed that its secretion plays an important role in maintaining feather integrity and plumage maintenance (see review in Moreno-Rueda, [44]). For example, Moreno-Rueda [45] showed a negative correlation between uropygial gland size and the number of feather holes caused by chewing lice in house sparrows. Similarly, Fülöp et al. [46] reported that the number of feather holes was negatively related to uropygial gland size during the breeding season in both male and female house sparrows. Since the number of feather holes is negatively correlated with feather quality [47], these results may suggest that gland secretion could promote feather quality by affording resistance against these ectoparasites. Also, other studies have revealed an antimicrobial capacity against feather-degrading bacteria in the uropygial secretion of some bird species such as hoopoe (*Upupa epops*) [48] and spotless starling (*Sturnus unicolor*) [49]. More recently, Bodawatta et al. [50] tested the potential defensive properties of uropygial gland bacteria from great tits (*Parus major*), showing that some of the bacterial isolates restricted the growth of feather-degrading bacteria. Since feather bacteria may provoke plumage degradation [51], the antimicrobial properties of uropygial secretion may improve feather quality. Nevertheless, studies directly analysing the relationships between uropygial gland volume and feather quality would be desirable. Moreover, it has been suggested that the uropygial gland may affect moult speed. In this sense, Moreno-Rueda [52] examined moult performance and uropygial gland size in house sparrows, showing that individuals with smaller uropygial glands had more feather holes, and those with more feather holes moulted later and faster. However, the number of studies exploring the relationship between uropygial gland secretion and moult duration is still scarce.

Here we first aim to determine whether avian haemosporidian infection influences feather quality and feather growth rate in two species of migratory passerines during the breeding season—the house martin (*Delichon urbicum*) and the sand martin (*Riparia riparia*). If avian haemosporidian infection negatively impacts both avian parameters, then we can expect that haemosporidian-infected birds should have lower feather quality and/or a lower feather growth rate than non-infected individuals. We also explored the role of the uropygial gland on the feather growth rate and feather quality in the two species of hirundines. If uropygial gland secretion positively influences plumage quality and moult performance, then we predict that individuals with smaller uropygial glands should have lower values of plumage quality and feather growth rate.

## 2. Material and Methods

### 2.1. Study Species

The house martin is a migratory passerine bird that breeds in the Palearctic region, from western Europe and North Africa to eastern Asia, and winters in Ethiopian region, across tropical and southern Africa to the sub-Saharan area, with a fairly large range of occurrence [53]. This is an insectivorous species closely associated with human environments, as it has an affinity for building its nests and forming numerous colonies in structures of anthropic origins such as buildings, bridges, and dams [54].

The sand martin is also a migratory species in the swallow family. It is widely distributed worldwide, breeding throughout temperate, boreal, and arctic latitudes of the Nearctic (North America) and Palearctic regions, from western Europe to the northern half of Asia and northern Japan. This insectivorous species winters in Ethiopian (eastern and southern Africa), Neotropical (across South America), and Oriental regions (Indian Subcontinent) [53]. Its seasonality in the Iberian Peninsula coincides with that of the house martin, although it is more specialized in building its nests, constructing tunnels in slopes resulting from fluvial erosion, but it has also been able to adapt to artificial substrates resulting from anthropic activities, such as gravel pits and quarries [55].

### 2.2. Study Area and Bird Sampling

**Figure 1:**
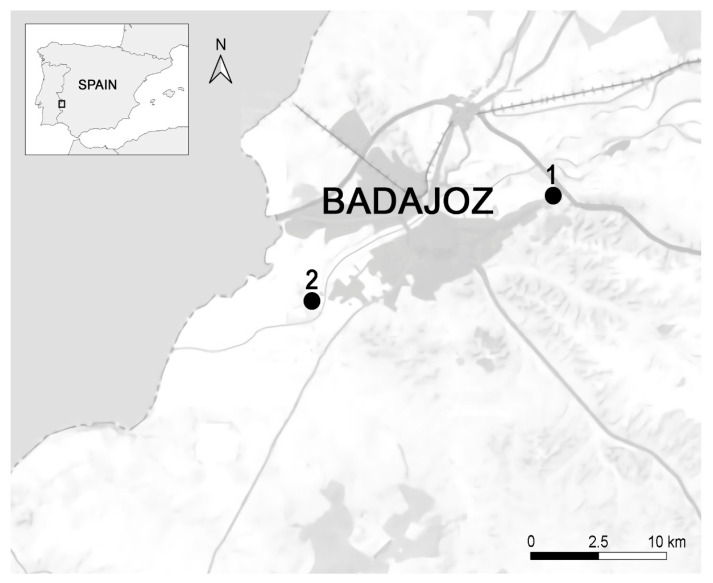
Distribution of the bird colonies of the study’s species. 1: house martins; 2: sand martins.

In late May and mid-June 2020, we used mist nets to capture 219 adult hirundinids in the province of Badajoz (Extremadura), southwestern Spain (Figure 1). House martins (*N* = 123) were captured on 12 June in one breeding colony located under a water tank (38°53′10.3″ N 6°55′32.3″ W). Sand martins (*N* = 96) were mist-trapped on 31 May at their nesting site in a sandy cliff of a sand pit close to the Guadiana River (38°51′28.3″ N 7°01′41.8″ W). Each bird was ringed with a numbered metal ring, and its age and sex were determined when possible, according to their plumage characteristics and skull ossification [56]. For each captured individual, we assessed body mass using a digital balance accurate to 0.1 g. Tarsus length was measured using a digital calliper with a precision of 0.01 mm. Subsequently, we estimated the scaled body mass index, a reliable metric for evaluating the physical condition of birds [57,58]. Additionally, we collected the second right outer rectrix feather from each individual and stored it in a plastic bag for subsequent estimations of feather growth rate and feather quality. During sampling, we did not collect feathers from individuals whose plumage were not fully developed or whose tails were in poor condition. Also, a blood sample was extracted from the jugular vein of each bird using sterile syringes and stored until molecular analysis. The volume of blood extracted from each individual was according to its body size and never exceeded 1% of its body mass. After manipulation, each bird was promptly released unharmed at its site of capture.

### 2.3. Measurement of Feather Growth Rate and Determination of Feather Quality Index

Feathers collected from all individuals were used to measure the feather growth rate and feather quality. Bird feathers exhibit a series of light and dark bands perpendicular to the feather rachis. Each light and dark band combination represents a growth bar, equivalent to approximately 24 h of growth [59,60,61,62]. Therefore, the number of dark bands indicates the number of days spent moulting these feathers. The number of growth bars and the length of the feather rectrix were measured using a gel documentation system (Bio-Rad Gel Doc XR + System), following the methodology outlined by Shawkey et al. [63]. Briefly, feathers were placed in a light cabinet to visualize the growth bars, with a ruler (0.1 mm accuracy) located nearby as a scale guide. Once optimal contrast and brightness conditions were achieved, digital images of the feathers were captured. These images were then post-processed using ImageJ software (Version 1.53e 2020) [64] to enhance lighting conditions for a clearer visualization of the growth bars. Using this software, the number of growth bars and the length of the rectrix were measured. Feather growth rate is reported as the average length of growth per day (mm/day) [39,40,41]. Feather mass was estimated with an analytical balance (Shimazdu AP225WD) to the nearest 0.0001 g. The ratio between feather mass and feather length served as an index of feather quality at an intraspecific level [36,40], as it reflects the density of structural elements and, hence, indicates feather durability [65].

### 2.4. Uropygial Gland Volume

A digital calliper with a precision of 0.01 mm was used to measure the length, height, and width of the uropygial gland. The volume of the uropygial gland was calculated by multiplying its length, height, and width [66], as this is known to be positively correlated with the volume of uropygial gland secretions [67,68]. Since the uropygial gland is a soft tissue [67,69], we conducted three measurements for each of its dimensions to assess repeatability [52,67,70].

### 2.5. Molecular Detection of Haemosporidian Infection

DNA from blood samples were extracted using the MAGMAX PATHOGEN RNA/DNA KIT (Applied Biosystems™, reference: 4462359). Diluted genomic DNA (25 ng/μL) was used as a template in a nested polymerase chain reaction (nested-PCR) to determine the presence or absence of haemosporidian infections following protocols described by Hellgren et al. [71]. Briefly, we used specific primer HaemNF1 (5′-CATATATTAAGAGAAITATGGAG-3′) and HaemNR3 (5′-ATAGAAAGATAAGAAATACCATTC-3′) in the first PCR, followed by two nested PCRs (Applied Biosystems™ SimpliAmp™ Thermal Cycler) to amplify *Haemoproteus* and *Plasmodium* genera using the primer pair HaemF (5′-ATGGTGCTTTCGATATATGCATG-3′) and HaemR2 (5′-GCATTATCTGGATGTGATAATGGT-3′), as well as amplify *Leucocytozoon*, using primers HaemFL (5′-ATGGTGTTTTAGATACTTACATT-3′) and HaemR2L (5′-CATTATCTGGATGAGATAATGGGC-3′). The amplification was evaluated by running 2.5 µL of the final PCR product on a 2% agarose gel. All PCR experiments contained one negative control (ddH_2_O) for every 8 samples and one positive control for *Haemoproteus*/*Plasmodium* and another one for *Leucocytozoon* for every 24 samples.

### 2.6. Statistical Analysis

We conducted Shapiro–Wilk tests to evaluate the normality of the data distribution of all continuous variables used in statistic models. A Chi-squared test was conducted to examine potential differences in haemosporidian prevalence among the two bird species. General linear models (GLMs) were employed to investigate the factors contributing to variation in the feather quality index for each bird species, separately. Predictor variables included scaled body mass index, haemosporidian infection status (uninfected or infected), sex, uropygial gland volume, and feather growth rate. Additionally, GLMs were used to investigate the effect of sex, scaled body mass index, infection status (uninfected or infected), feather quality rate, and uropygial gland volume on the feather growth rate for each bird species separately. There were no significant correlations among the predictor variables (Pearson correlation, all *p* > 0.05); hence, they were included in the models as independent variables. Given the normality of the data, the Gaussian family was selected. Additionally, the adequacy of the models was assessed by examining their explained variances. To evaluate correlation between feather growth rate and feather quality, we calculated the Pearson correlation coefficient for each bird species, stratifying by infection status. All statistical analyses were carried out with R software version 4.2.2 [72].

## 3. Results

### 3.1. Haemosporidian Prevalence

Out of the 219 adult birds captured, 40 individuals showed avian haemosporidian infection, of which 32 (14.61%) corresponded to *Haemoproteus*/*Plasmodium* infections and 12 (5.48%) corresponded to *Leucocytozoon* infections (overall prevalence = 18.26%, 95% C.I. = 0.137–0.239). Specifically, out of the 123 house martins (75 males, 48 females), 25 (20.33%) were infected with *Haemoproteus*/*Plasmodium*, and 11 (8.94%) were infected with *Leucocytozoon*; while out of the 96 sand martins (43 males, 53 females), 7 (7.29%) were infected with *Haemoproteus*/*Plasmodium*, and only 1 (1.04%) was infected with *Leucocytozoon*. The haemosporidian prevalence differed among bird species (Chi-square test: *χ*^2^ = 10.14, d.f. = 1, *p* < 0.05). Thus, the prevalence of infection was higher in house martins (*N* = 123; prevalence = 26.02%; 95% C.I. = 0.004–0.333) than in sand martins (*N* = 96; prevalence = 8.33%; 95% C.I. = 0.043–0.156). Of the total number of birds, 1.83% (95% C.I. = 0.007–0.046) were coinfected with *Haemoproteus* or *Plasmodium* and *Leucocytozoon* parasites; these coinfections were only found in 4 house martins.

### 3.2. Factors Explaining Variation in Feather Quality Index

**Figure 2:**
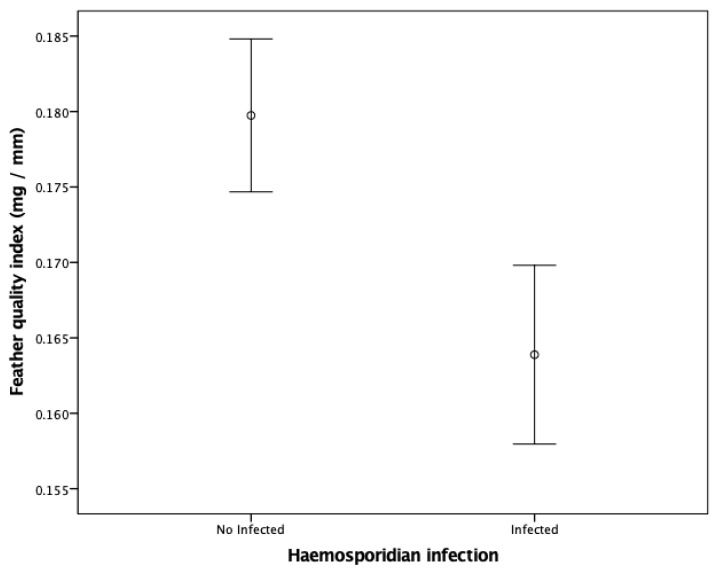
Feather quality index (mg/mm) for uninfected (*N* = 91) and infected (*N* = 32) house martins. Error bar plots show means ±95% confidence interval.

**Figure 3:**
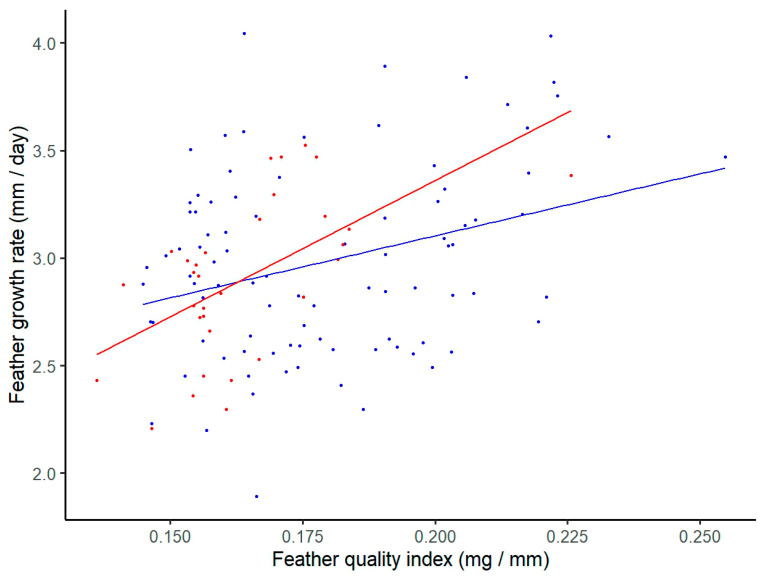
Scatter plot showing the relationship between the feather growth rate (mm/day) and feather quality (mg/mm) for uninfected (blue circle, *N* = 91) and infected house martins (red circle, *N* = 32).

All variables included in the GLMs showed normal distribution (*p* > 0.05). We found that only haemosporidian infection and feather growth rate significantly explained variation in feather quality in house martins (Table 1). Specifically, haemosporidian-infected birds exhibited lower feather quality values than uninfected individuals (mean feather quality index (SD): uninfected = 0.180 (0.024) mg/mm; infected = 0.164 (0.016) mg/mm) (Table 1, Figure 2). In addition, we found a significant positive correlation between the quality of the feather and the feather growth rate for both infected (Pearson correlation, r = 0.570, *p* < 0.05) and uninfected (Pearson correlation, r = 0.316, *p* < 0.05) house martins (Figure 3). By contrast, none of the predictors significantly explained variation in feather quality in sand martins (Supplementary Table S1, all *p* > 0.05).

### 3.3. Factors Explaining Variation in Feather Growth Rate

We found that only feather quality significantly explained variation in the feather growth rate in house martins (Table 2, Figure 3). Additionally, we observed a non-significant positive trend in the relationship between feather growth rate and feather quality for both infected (Pearson correlation, r = 0.478, *p* = 0.231) and uninfected (Pearson correlation, r = 0.159, *p* = 0.171) sand martins (Supplementary Figure S1). None of the other predictors (avian haemosporidian infection, sex, scaled body mass index, and uropygial gland volume) significantly influenced feather growth rate in sand martins (all *p* > 0.05) (Supplementary Table S2).

## 4. Discussion

Feathers serve birds not only as a means of flight but also by fulfilling other vital functions such as providing a protective barrier, insulation, aiding in communication among conspecifics, and camouflage [1]. Throughout the annual cycle, the continuous degradation of plumage requires birds to regularly replace worn feathers to maintain these functions and improve their fitness. However, environmental conditions experienced during moulting, including factors such as food availability, stressors, and pathogen infection, may impair feather quality and the growth rate of newly produced feathers [7]. In addition, it has been proposed that uropygial gland secretion may improve feather quality and reduce moult duration [46,52]. Here, we investigated whether avian haemosporidian infection and uropygial gland volume influence the feather quality and feather growth rate of the second outermost tail feathers in three species of migratory hirundinids during the breeding season in southwest Europe. Our main findings showed that (i) the prevalence of avian haemosporidian infection varied significantly among bird species; (ii) house martins infected with haemosporidian parasites exhibited lower feather quality; (iii) feather quality was positively correlated with feather growth rate in house martins; (iv) avian haemosporidian infection did not affect feather growth rate in any of the analysed bird species; and (v) no relationship was found between uropygial gland volume and feather quality or feather growth rate in any of the studied species.

### 4.1. Differences in Haemosporidian Prevalence between Bird Species

Our findings revealed significant variations in avian haemosporidian infection among the two hirundine species studied. This aligns with previous research across different avian taxonomic orders, which also reported differences in haemosporidian prevalence among closely related bird species. For instance, Inumaru et al. [73] found variations in the prevalence of infection with *Plasmodium* and *Haemoproteus* across five species of *Gallinago* snipes in Japan. Similarly, Dubiec et al. [74] observed differential prevalence rates of haemosporidian parasites (i.e., *Plasmodium* and *Haemoproteus*) in nest-box breeding populations of great tits (*Parus major*) and blue tits (*Cyanistes caeruleus*) in Southern Gotland, Sweden, with great tits exhibiting higher infection rates than blue tits. Moreover, Ellis et al. [75] analysed the haemosporidian prevalence in Neotropical birds, showing variations among taxonomic families, genera, and even species within the same genus. Recently, Bukauskaité et al. [76] examined the prevalence of haemosporidian parasites in two sympatrically breeding species of the order Accipitriformes from temperate forests of central–eastern Europe, reporting significantly lower infection rates in the white-tailed eagle *Haliaeetus albicilla* compared to the lesser spotted eagle *Clanga pomarina*.

Our study revealed a higher prevalence of infection in house martins compared to sand martins. Such interspecific variation in haemosporidian parasite prevalence is often attributed to differences in vector exposure [77,78]. For example, open-nesting bird species are more susceptible to haemosporidian infections than closed-nesting species or birds nesting in cavities, probably due to increased vector detection [79,80,81]. However, the inter-individual transmission of pathogens is assumed to increase linearly with host density [82], potentially explaining the higher prevalence observed in house martins due to their larger colony size [83,84]. In our study, the number of breeding pairs in the house martin colony was higher than the number of pairs nesting in the sand martin colony, which may explain the higher haemosporidian prevalence observed in house martins. Although sand martins also breed in substantial numbers, their nesting behaviour, with eggs laid in tunnels up to a meter long [85], may limit vector exposure [86]. In fact, the abundance and diversity of mosquitoes in the area of the sand martin colony are notably higher than in the area of the house martin colony [87], which seems to support this latter idea.

Alternatively, environmental conditions at wintering and stopover sites may also influence the likelihood of migratory species becoming infected with haemosporidians. For example, it is known that populations of sand martins from western Europe (Great Britain, Spain, and Portugal) migrate to wintering areas with a sub-Saharan desert climate located in the Senegal River Delta [88], while house martin populations from southwest Spain winter in higher rainfall habitats such as the west African broadleaf forests [89], potentially exposing them to higher vector densities [90]. This, in turn, can increase their probability of acquiring haemosporidian infections [91]. Finally, the interplay between host immune defences and parasite exploitation strategies may further influence haemosporidian prevalence in bird communities [92].

### 4.2. Factors Influencing Feather Quality Index 

Several studies have shown that, among other stressors, parasites can impact the feather quality of birds. For example, Pap et al. [37] observed that house sparrows receiving anticoccidial treatment developed larger and heavier primaries with increased vane area and thicker rachis compared to untreated conspecifics, thus revealing that coccidian infestation reduces the quality of the flight feathers. Similarly, Pérez-Tris et al. [36] reported that feather quality significantly decreased with mite infestation intensity in fledgling blackcaps (*Sylvia atricapilla*). According to our predictions, we observed reduced feather quality in house martins infected with haemosporidians. To our knowledge, this is the first study revealing the negative impact of haemosporidian infection on the feather quality of bird hosts. We propose two non-mutually exclusive hypotheses to explain these results.

First, parasites may compete with their bird hosts for resources. Studies have shown that haemosporidian parasites are unable to de novo synthetize certain amino acids required for their growth and development, such as isoleucine and methionine, which must be acquired from their hosts [93]. Since both methionine and isoleucine are involved in synthesizing feather keratin and are crucial for the feather growth [94,95], a deficiency in these critical feather constituents resulting from pathogen consumption may impair the production of high-quality feathers.

Second, given that both the activation of an immune response to face the pathogen challenge (e.g., haemosporidian infection) and the production of high-quality feathers are energetically and nutritional demanding processes [7,96], a trade-off in resource allocation between these two traits is expected. Supporting this notion, Ben-Hamo et al. [97] reported reduced quality in newly grown feathers after an immune challenge in house sparrows, suggesting that the allocation of resources to mounting an immune response may compete with feather growth. However, haemosporidian infection did not affect feather quality in sand martins. Because of the low number of haemosporidian-infected birds sampled of this species, further studies with larger sample sizes are needed to assess whether haemosporidian parasites have any effect on feather quality in this hirundine. Also, due to the low number of infected birds, we grouped the infections of all haemosporidian genera to test for the effect of overall infection on feather quality and feather growth rate. As different haemosporidian genera may show different effects on their hosts [98,99], further studies with larger sample sizes are required to separately investigate the effects of distinct parasite genera.

Some authors have pointed out that rapid feather growth may led to poor feather quality [40,100], thus suggesting a trade-off between feather growth rate and feather quality but resulting in the contradiction of high-quality individuals producing feathers of poor quality, which could lead to decreased fitness [7]. However, an increasing body of literature has revealed that, within populations, individuals with high feather growth rates tend to exhibit higher overall quality [101,102,103,104]. In addition, feather quality has been linked to good individual body conditions in great tits [16] and house sparrows [105], further supporting the idea that individuals in good conditions generally produce feathers of a higher quality at a faster rate [7].

Our results align with this perspective, showing a positive correlation between feather quality and feather growth rate in house martins. Similar patterns have been observed in other species, such as great tits, where a positive relationship between feather growth rate and feather mass has been documented [106]. Nonetheless, this association was not as straightforward in sand martins. Studies have also identified differences in the relation between feather growth rate and feather quality among species [107], or even within populations of the same species [101]. These differences are often attributed to differences in resource availability during the feather renewal period or time constraints imposed by the potential overlap of annual cycle activities [7,101].

It has been proposed that uropygial secretion plays a role in protecting and maintaining the plumage by shielding it from various external agents, including solar radiation, abrasion, and ectoparasites, among others [44,45,46]. Therefore, given that larger uropygial glands produce more secretion [69], a positive relationship between plumage quality and uropygial gland volume was to be expected. Yet, no relationship was found between feather quality and uropygial gland volume in either of the two hirundine species. This discrepancy could be attributed to variations in the quantity and composition of uropygial secretion, which can vary not only among different bird species [108] but also seasonally within the same species [68,69,109]. Therefore, both inter- and intraspecific variations in the quantity and composition of uropygial secretion, as well as seasonality, may influence its capacity to protect the plumage and maintain its quality. Despite these observations, further research efforts are needed to understand the role of the uropygial gland and its secretion in the production of plumage and maintenance of feathers of optimal quality.

### 4.3. Factors Influencing Feather Growth Rate 

While numerous studies have explored factors potentially influencing the feather regrowth rate by experimentally plucking feathers, limited data exist on environmental effects on the naturally moulting feather growth rate (see review in [7]). Here, we assessed the association between the growth rate of naturally moulted feathers and haemosporidian infection in two hirundine species. Contrary to our predictions, the feather growth rate was not related to haemosporidian infection in any of the studied bird species. These results contrast with observational and experimental studies analysing the feather growth rate and haemosporidian infection in house martins [39,40] and house sparrows [41] from the same geographical area in previous study years. Similarly, previous studies have also shown contrasting results between study years on the effect of parasite infections on feather quality and feather growth rate. For example, Pap et al. [37] conducted experimental research on house sparrows in two consecutive moults, showing that coccidian infection significantly reduced the stiffness of the feather grown after the first moult but not in the case of feathers grown after the second moult. Also, Dunn et al. [110] investigated the potential for haemosporidian parasites to impact the feather growth of yellowhammers (*Emberiza citrinella*), revealing that birds infected with haemosporidians had a shorter feather growth rate in the winter of 2007/2008 but not in the winter of 2008/2009. Overall, these inter-annual differences suggest a year-dependent association between parasite infection and reduced feather quality and/or feather growth rate, indicating that the effects of haemosporidian infection on these parameters are still poorly explored and deserve more attention in further studies.

Moreover, other previous studies have also failed to detect the negative effect of haemosporidians on the feather growth rate. For example, Romano et al. [111] investigated haemosporidian infection in adult barn swallows and its consequences on the feather growth rate, showing a negative effect of infection by *Plasmodium* on the feather growth rate in older individuals but not in yearlings. While all the sampled individuals in our analyses were adults, we unfortunately did not categorize them into different age classes. Further studies would now be required to disentangle the potential age-dependent differences in the effect of haemosporidian infections on the feather growth rate.

Also, Henschen et al. [112] analysed plumage ornaments and parasite infection in common yellowthroat (*Geothlypis trichas)* males, revealing that neither the presence nor the intensity of haemosporidian infection was related to the feather growth rate. In this latter study, most haemosporidian infections were chronic, low-intensity infections (the percent of red blood cells infected was less than 2%) rather than acute infections, which usually show few or no measurable harmful effects on their hosts [98,113,114,115]. Because the negative effects of haemosporidians on the host phenotype usually occur when parasitaemia reaches higher levels (higher than 2% of infected erythrocytes), typically during a short initial primary infection or in relapses [23,25], experimental infections with haemosporidians inducing initial acute parasitaemia would be fundamental to reliably show the potential negative effects of haemosporidian infection on the feather growth rate of their avian hosts.

Finally, it has been shown that individuals in better conditions exhibit faster feather growth [41,103]. Because birds with better body conditions normally produce larger uropygial glands [45,70,116,117], a positive relationship between uropygial gland size and feather growth rate could be predicted. However, we did not observe any relationship between the feather growth rate and the size of the uropygial gland. Moreover, Møller and Laursen [118] found that eiders (*Somateria mollisima*) with small uropygial glands grew their feathers at a faster rate. In this regard, the uropygial gland may not be related to high feather quality and higher feather growth rates. Instead, larger glands might be necessary when feathers are subjected to increased damage or when optimal growth is limited. Therefore, uropygial secretion may not suffice to compensate for detrimental effects, which could lead to the observation of individuals with large glands but suboptimal feathers. Further investigations are needed to confirm whether the size of the uropygial gland might affect the feather growth rate.

## 5. Conclusions

This study appraised the prevalence of avian haemosporidian infection and its impact on the plumage of two species of hirundinids in southwestern Europe. The prevalence of haemosporidian parasites varied among species, with the highest rate observed in house martins, possibly due to the size of their colonies. Additionally, avian haemosporidian infection was found to have adverse effects on the feather quality of this hirundine, representing the first evidence of this effect of haemosporidian parasites on this parameter. However, the infection did not appear to influence the feather quality of sand martins. Likewise, there was no observed relationship between haemosporidian infection and the feather growth rate, although future studies with larger sample sizes could provide more conclusive evidence of these effects. Moreover, a positive correlation was observed between feather quality and moulting speed in house martins, supporting the notion that high-quality or well-conditioned individuals tend to produce high-quality feathers and moult them more quickly. Finally, we found no relationship between the volume of the uropygial gland and the feather quality or feather growth rate in any species. These findings contribute to our understanding of the impact of haemosporidian infections on the plumage of migratory birds, although further research is required to explore these relationships more comprehensively.

## Figures and Tables

**Table 1:** Results from the GLM explaining variation in feather quality (mg/mm) for house martins (*N* = 123). Haemosporidian infection, sex, scaled body mass index, uropygial gland volume (mm^3^), and feather growth rate (mm/day) were included as predictor variables. Significant factors are highlighted in bold.

Independent Variables	Estimate	Std. Error	*t*	*p*
Haemosporidian infection (infected)	−0.043	0.014	−3.060	**0.003**
Sex	0.006	0.011	0.495	0.622
Scaled body mass index	−0.269	0.197	−1.363	0.176
Uropygial gland volume	<0.001	<0.001	0.137	0.891
Feather growth rate	0.262	0.087	3.004	**0.004**

**Table 2:** Results from the GLM explaining variation in feather growth rate (mm/day) for house martins (*N* = 123). Haemosporidian infection, sex, scaled body mass index, uropygial gland volume (mm^3^), and feather quality (mg/mm) were included in the analysis as predictor variables. Significant factors are highlighted in bold.

Independent Variables	Estimate	Std. Error	*t*	*p*
Haemosporidian infection (infected)	0.004	0.018	0.219	0.827
Sex	−0.014	0.013	−1.011	0.315
Scaled body mass index	0.359	0.234	1.535	0.129
Uropygial gland volume	−0.001	0.001	−1.627	0.107
Feather quality	0.370	0.123	3.004	**0.004**

## Data Availability

The authors confirm that the data supporting the findings of this study are available within the article and in Supplementary Tables S1 and S2.

## Supplementary Materials

The following supporting information can be downloaded at https://www.mdpi.com/article/10.3390/ani14121772/s1. Figure S1: Scatter plot showing the relationship between the feather growth rate (mm/day) and feather quality (mg/mm) for uninfected (blue symbol, *N* = 88) and infected sand martins (red symbol, *N* = 8); Table S1: Results from the GLM explaining variation in feather quality (mg/mm) for sand martins (*N* = 96). Haemosporidian infection, sex, scaled body mass index, uropygial gland volume (mm3) and feather growth rate (mm/day) were included in the analysis as predictor variables; Table S2: Results from the GLM explaining variation in feather growth rate (mm/day) for sand martins (*N* = 96). Haemosporidian infection, sex, scaled body mass index, uropygial gland volume (mm3) and feather quality (mg/mm) were included in the analysis as predictor variables.
