# Supplementary material for: The Impact of Avian Haemosporidian Infection on Feather Quality and Feather Growth Rate of Migratory Passerines
Source: Animals (Basel). 2024 Jun 12;14(12):1772. doi: 10.3390/ani14121772 (PMC11200494; doi:10.3390/ani14121772)
Supplement: Supplementary file 1 [file animals-14-01772-s001.zip › animals-3020532-supplementary.pdf]

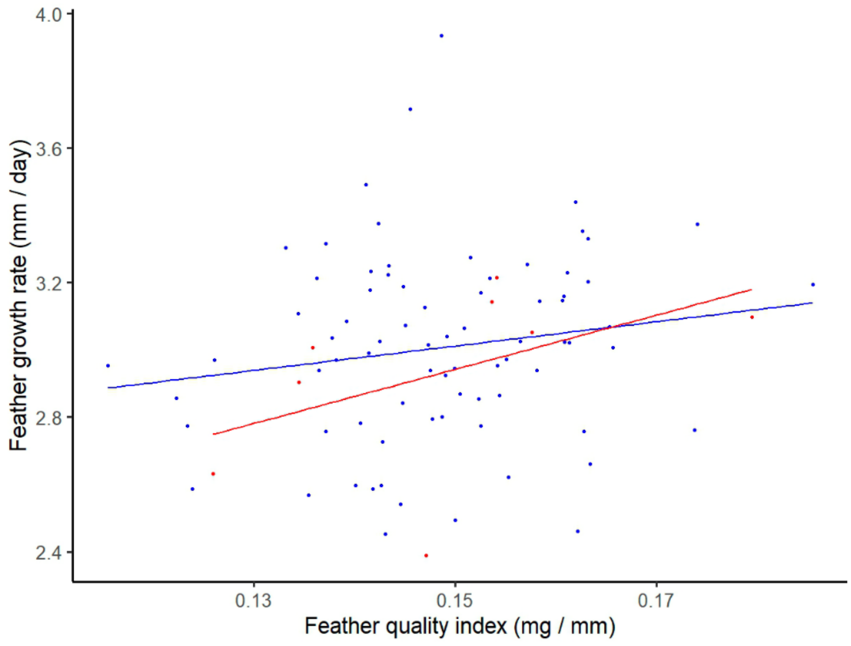

Figure S1. Scatter plot showing the relationship between the feather growth rate (mm / day) and feather quality (mg / mm) for uninfected (blue symbol, N = 88) and infected sand martins (red symbol, N = 8).

Table S1. Results from the GLM explaining variation in feather quality (mg / mm) for sand martins (N = 96). Haemosporidian infection, sex, scaled body mass index, uropygial gland volume (mm^3) and feather growth rate (mm / day) were included in the analysis as predictor variables.

Independent variables	Estimate	Std. Error	*t*	*p*
Haemosporidian infection (infected)	0.003	0.014	0.181	0.857
Sex	-0.006	0.012	-0.480	0.633
Scaled body mass index	-0.067	0.135	-0.498	0.620
Uropygial gland volume	<0.001	<0.001	0.910	0.366
Feather growth rate	0.183	0.103	1.774	0.080

Table S2. Results from the GLM explaining variation in feather growth rate (mm / day) for sand martins (N = 96). Haemosporidian infection, sex, scaled body mass index, uropygial gland volume (mm^3) and feather quality (mg / mm) were included in the analysis as predictor variables.

Independent variables	Estimate	Std. Error	*t*	*p*
Haemosporidian infection (infected)	-0.012	0.015	-0.789	0.432
Sex	0.018	0.013	1.384	0.170
Scaled body mass index	0.084	0.146	0.577	0.566
Uropygial gland volume	<0.001	<0.001	0.071	0.944
Feather quality	0.213	0.120	1.774	0.080
